# Regulation of the Homeostatic Unfolded Protein Response in Diabetic Nephropathy

**DOI:** 10.3390/ph15040401

**Published:** 2022-03-25

**Authors:** Hongjie Wang, Srikanth Karnati, Thati Madhusudhan

**Affiliations:** 1 Division of Cardiology, Department of Internal Medicine, Tongji Hospital, Tongji Medical College, Huazhong University of Science and Technology, Wuhan 430030, China; hongjie.wang@tjh.tjmu.edu.cn; 2 Hubei Key Laboratory of Genetics and Molecular Mechanisms of Cardiological Disorders, Wuhan 430030, China; 3 Institute of Anatomy and Cell Biology, Julius-Maximilians-University Würzburg, 97070 Würzburg, Germany; srikanth.karnati@uni-wuerzburg.de; 4 Center for Thrombosis and Hemostasis, University Medical Center Mainz, Langenbeckstr. 1, 55131 Mainz, Germany

**Keywords:** unfolded protein response, ER stress, diabetic nephropathy, insulin signaling, aPC, podocytes, XBP1, ATF6

## Abstract

A growing body of scientific evidence indicates that protein homeostasis, also designated as proteostasis, is causatively linked to chronic diabetic nephropathy (DN). Experimental studies have demonstrated that the insulin signaling in podocytes maintain the homeostatic unfolded protein response (UPR). Insulin signaling via the insulin receptor non-canonically activates the spliced X-box binding protein-1 (sXBP1), a highly conserved endoplasmic reticulum (ER) transcription factor, which regulates the expression of genes that control proteostasis. Defective insulin signaling in mouse models of diabetes or the genetic disruption of the insulin signaling pathway in podocytes propagates hyperglycemia induced maladaptive UPR and DN. Insulin resistance in podocytes specifically promotes activating transcription factor 6 (ATF6) dependent pathogenic UPR. Akin to insulin, recent studies have identified that the cytoprotective effect of anticoagulant serine protease-activated protein C (aPC) in DN is mediated by sXBP1. In mouse models of DN, treatment with chemical chaperones that improve protein folding provides an additional benefit on top of currently used ACE inhibitors. Understanding the molecular mechanisms that transmute renal cell specific adaptive responses and that deteriorate renal function in diabetes will enable researchers to develop new therapeutic regimens for DN. Within this review, we focus on the current understanding of homeostatic mechanisms by which UPR is regulated in DN.

## 1. Regulation of Unfolded Protein Response and ER Stress

Within the endoplasmic reticulum (ER), a high demand for proper protein synthesis and folding leads to the accumulation of misfolded proteins, resulting in ER stress and thus triggering the unfolded protein response (UPR) [1]. ER stress can be induced by physiological stress associated with organ development or aging and by pathogenic stress associated with the development and progression of various diseases, including kidney disease [2]. Protein misfolding and the activation of ER stress has been reported in various human and experimental models of renal diseases, including primary glomerulonephritides, glomerulopathies associated with genetic mutations, renal fibrosis, acute kidney injury, and chronic kidney diseases such as diabetes induced nephropathy (DN) [3]. Proteostasis defects that arise from ER folding deficiencies are at the core of metabolic syndrome, diabetes, and diabetes-associated chronic complications [4]. In the leptin deficient ob/ob mice, obesity and insulin resistance (IR) are ameliorated by enhancing ER-protein folding capacity using the chemical chaperones 4-phenylbutarate (4-PBA) and taurine-conjugated ursodeoxycholic acid (TUDCA) [5]. Subsequently, the beneficial effects of these chemical chaperones were demonstrated in several mouse models of DN [3]. These initial studies provided experimental evidence that inhibition of ER stress is a promising approach for the treatment of DN. However, the underlying molecular mechanisms and the specific UPR pathways that are causatively linked to DN remain unclear. Experimental studies delineating the activation of UPR pathways typically use chemical inducers that interfere with the regulation of protein folding processes. These include tunicamycin (blocks N-linked glycosylation), thapsigargin (inhibitor of Sacrco/endoplasmic Ca^2+^-ATPases), and brefeldin A (inhibits protein translocation from ER to the Golgi-complex). Studies using these compounds provided the conceptual framework that enabled researchers to identify the specific UPR pathways and the downstream regulators that either restore ER homeostasis or promote ER stress induced cell death [6]. However, unlike these compounds that activate all the major pathways of UPR, the regulation of UPR in a disease state is distinct and it may involve aberrant activation or inactivation of one of the major pathways [7,8,9]. This could be due to the disease specific loss of protective factors that govern homeostatic UPR or induction of specific pathogenic stimuli as a result of intercellular crosstalk, an altered tissue microenvironment, and systemic inflammation. Therefore, understanding the predominant disease-specific regulation of UPR and their physiological regulators is essential for developing targeted therapies. Within the current review, we elaborate on major pathways that are differentially regulated in DN with a special focus on the homeostatic mechanisms that preserve UPR-ER in the kidney. For additional mechanisms of ER stress and UPR in other acute and fibrotic kidney diseases, we refer to the recent excellent review [3].

**Figure 1:**
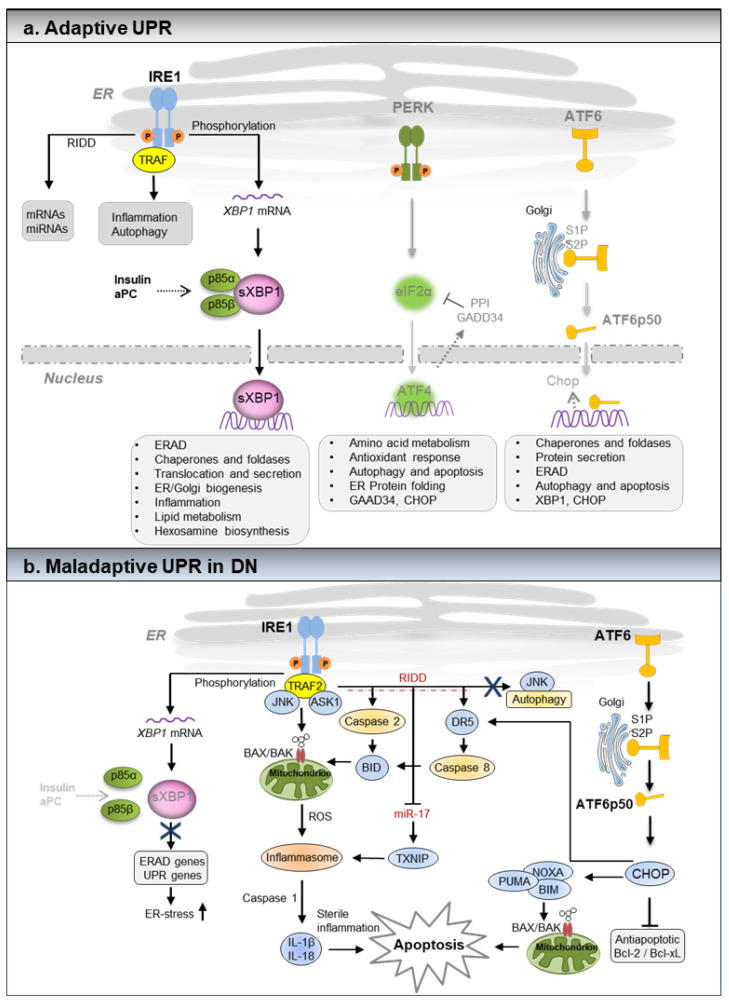
(**a**,**b**): Scheme showing adaptive (homeostatic) and maladaptive unfolded protein response (UPR) in diabetic nephropathy (DN). Adaptive UPR: Accumulation of misfolded proteins within the ER causes ER stress, which is sensed by the three major ER-transmembrane proteins IRE1, PERK, and ATF6. IRE1 pathway: Upon activation, IRE1 through its endoribonuclease activity induces the splicing of ER-transcription factor XBP1. The spliced XBP1 (sXBP1) translocates to the nucleus and induces the expression of genes that encode proteins involved in mitigation of ER stress. Independent of IRE1 activation, the nuclear translocation of sXBP1 is mediated by insulin and coagulation protease activated protein C (aPC). Within the insulin sensitive renal podocytes, insulin signaling via insulin receptor and the downstream regulatory subunits of PI3Kinase, p85α, and p85β promotes nuclear translocation of sXBP1. Akin to insulin, aPC signaling via protease activated receptors promotes nuclear translocation of sXBP1 in a p85α and a p85β dependent fashion. Both insulin and aPC signaling in glomerular cell types (podocytes and endothelial cells) specifically activates sXBP1 dependent proteostasis pathway, without inducing PERK or ATF6 pathways. During severe or unmitigated ER stress, IRE1α-RNase can also cleave ER-localized mRNAs or non-coding functional RNAs, leading to their degradation through regulated IRE1-dependent decay (RIDD). RIDD not only reduces the ER protein folding load, but also modulates inflammation, metabolism, and inflammasome signaling pathway. Additionally, the cytoplasmic domain of IRE1α serves as a scaffold, which recruits adaptor proteins (e.g., tumor necrosis factor receptor-associated factor (TRAF). The IRE1/TRAF complexes activate inflammatory responses under non-canonical ER stress conditions. PERK pathway: Upon activation, PERK induces the phosphorylation of eIF2α which transiently attenuates the translation in order to reduce protein synthesis. Concomitant activation of stress inducible transcription factor ATF4 induces the expression of genes that are involved in redox homeostasis, amino acid metabolism, autophagy, ER-protein folding, and apoptosis. Additionally, ATF4 participates in a feedback loop mediated by PPI and GADD34 to dephosphorylate eIF2α and to restore protein synthesis. However, severe ER stress may trigger ATF4-dependent cell death program by activation of CHOP. Thus, the magnitude of ER stress and the disease-specific stimuli may disparately modulate PERK-ATF4 dependent adaptive and maladaptive cellular responses. ATF6 pathway: Upon activation ATF6 relocates to the Golgi-complex. Within the Golgi-complex the full length ATF6α-p90 undergoes proteolytic cleavage mediated by site1 and site2 proteases (S1P and S2P), resulting in a highly active transcription factor ATF6α-p50, which translocates to the nucleus to induce gene expression. ATF6 induces the expression of genes that are involved in ER-protein folding, protein secretion, ERAD, autophagy, and apoptosis. Figure 1b: Maladaptive UPR in diabetic nephropathy (DN). The disease-specific stimuli (for e.g., hyperglycemia) in chronic DN coupled with loss of insulin or insulin resistance diminishes the nuclear translocation of sXBP1. Subsequently, chronic hyperglycemia specifically activates ATF6 pathway, which promotes the expression of proapoptotic CHOP and DN. Of note, activation of the PERK-ATF4 pathway was not observed in DN, suggesting cell- and disease-specific regulation of the UPR. The loss of the sXBP1 function often results in hyperactivation of IRE1α. The IRE1α-TRAF2/JNK and IRE1α-RIDD axis activates BAX/BAK mediated mitochondrial dysfunction and apoptosis by activating caspase-2 or caspase-8 dependent activation of BH3-interacting domain death agonist (BID). Additionally, the IRE1α-RIDD axis promotes degradation of microRNA-17(miR-17), which stabilizes the thioredoxin-interacting protein (TXNIP) and promotes inflammasome activation. Inflammasome activation mediated by TXNIP or mitochondrial reactive oxygen species (ROS) promotes caspase-1/IL-1β/IL-18 dependent sterile inflammation. The IRE1α-TRAF2/JNK signaling also modulates autophagy. Additionally, persistent activation of ATF6α in DN may promote a CHOP-dependent apoptosis program. CHOP promotes ER stress induced apoptosis by activating death receptor 5 (DR5) dependent caspase-8 activation or by activating the Bcl-2 and BH3-only family members NOXA, PUMA, BID, BAX, and BAK.

The UPR is the central homeostatic regulator of ER function, as has been shown by the pioneers Kazu Mori, Peter Walter, and coworkers in the early 1990s [10,11]. In metazoans, the UPR is mediated by the three ER-localized sensors: inositol requiring-enzyme 1 (IRE1), PKR-like ER Kinase (PERK), and activating transcription factor 6 (ATF6) [8,12] (Figure 1a). In non-stressed cells, these transmembrane proteins are bound to the ER chaperone: binding immunoglobulin protein 1 (BIP) (also known as glucose regulated protein 78 (GRP78) or HSPA5) [8,12]. The BIP is the master regulator that plays an essential role in the sensing and the activation of the ER-UPR [13]. Upon ER stress, BIP/GRP78 is released from the ER-transmembrane signal transducers IRE1, PERK, and ATF6, leading to the activation of downstream UPR signaling pathways (Figure 1a). Homozygous BIP knockout mice demonstrate lethality by embryonic day 3.5 with a much reduced proliferation rate of embryonic cells and massive apoptotic death of the inner cell mass, making it difficult to interpret the renal-specific function of the BIP [13]. However, the role of the BIP and ER stress in renal cells was examined in a knock-in mice which expresses a mutant BIP that lacks the ER-retention sequence. While homozygous BIP-mutant mice showed signs of ER stress, and they died shortly after birth, heterozygous BIP-mutant mice showed significant tubular-interstitial lesions with aging [14]. Akin to the BIP, homozygous deletion of ER-transmembrane signal transducers IRE1α and PERK, and a combined deletion of ATF6α and ATF6β are also embryonically lethal. However, subsequent studies in renal cell-specific deletion mutants or in glomerulopathies associated with genetic mutations revealed the critical role of these UPR regulators in several kidney diseases including DN [3].

## 2. IRE1 Pathway

The IRE1 pathway contains an ER luminal sensor domain and a cytosolic endoribonuclease (RNase) domain. Upon activation and dissociation from the BIP in response to ER stress, IRE1 oligomerizes and transphosphorylates neighboring IRE1 molecules. This results in the activation of its endoribonuclease activity, which catalyzes the unconventional splicing of the mRNA encoding the transcription factor, X- box- binding protein 1 (XBP1) [15,16,17]. The IRE1-dependent activation of spliced XBP1 (sXBP1) plays a critical role in maintaining ER-proteostasis by regulating the expression of multiple UPR target genes encoding proteins involved in ER protein folding, translocation, secretion, ER-associated degradation (ERAD), ER and Golgi biogenesis, and inflammation (Figure 1a) [18]. On the other hand, severe or prolonged ER stress increases the IRE1 endoribonuclease activity, which results in cleavage of additional ER-localized mRNAs and microRNAs (miRNAs) through a process known as the “Regulated IRE1-dependent decay (RIDD) [19,20]. The IRE1 dependent RIDD requires a common consensus CUGCAG sequence motif within a stem-loop for both XBP1 and RIDD-regulated mRNAs and precursor miRNAs. The IRE1 pathway also modulates apoptosis and autophagy by activating the c-Jun amino-terminal kinases (JNK or SAPKs) or the pro-apoptotic BCL2 family members BAX and BAK. The interaction of the cytoplasmic domain of IRE1 with the adaptor protein TNF receptor associated factor 2 (TRAF2) couples plasma membrane receptors to JNK activation [21], whereas the interaction of IRE1 cytoplasmic domain with BAX and BAK modulates IRE1 activation [22]. Thus, IRE1 is a multifunctional protein that primarily aims to restore protein homeostasis through various transcriptional, post-transcriptional, and scaffolding mechanisms. However, prolonged or unmitigated ER stress in chronic diabetes may activate inflammasome and proapoptotic responses through RIDD or activation of TRAF2-JNK signaling [8,23,24], (Figure 1b). Podocyte-specific deletion of IRE1α results in albuminuria in male mice beginning at 5 months of age. Podocyte foot process effacement as well as microvillous transformation of podocyte plasma membranes was observed in 9-month-old male mice with podocyte-specific IRE1α-deletion [25]. Unlike IRE1α, podocyte-specific deletion of XBP1 showed no signs of glomerular injury in mice up to 12 months of age [26]. However, XBP1 deletion in podocytes exacerbated ER stress and glomerular injury in a streptozotocin model of DN [27]. Additionally, combined podocyte-specific deletion of XBP1 and Sec63, a heat shock protein-40 chaperone required for protein folding in the ER, caused apoptosis of podocytes, glomerulosclerosis, and progressive albuminuria by 8–10 weeks of age [26].

## 3. PERK Pathway

An additional mechanism through which UPR engages in resolving unabated ER stress is by translational attenuation, controlled by the PERK-activating transcription factor 4 (ATF4) pathway. Upon activation, UPR transducer PERK mediates the phosphorylation of the eukaryotic translation initiation factor 2 alpha (eIF2α), which leads to general translational attenuation, while selectively activating the expression of ATF4, and subsequent induction of the pro-apoptotic transcription factor C/EBP homologous protein (CHOP) [20]. Often, the translational attenuation mediated by the phosphorylated eIF2α is a reversible process in which ATF4 participates in a feedback loop to dephosphorylate eIF2α, which restores protein synthesis through the upregulation of protein phosphatase 1 (PP1) regulatory subunit, growth arrest, and DNA damage-inducible protein-34 (GADD34) (Figure 1a) [28,29,30]. In addition to PERK, a few other stress-responsive kinases including protein kinase R (PKR), heme-regulated eIF2α kinase (HRI), and general control nonderepressible 2 (GCN2) can also drive the phosphorylation of eIF2α. The effector program downstream of the transcription factors ATF4, activating transcription factor 5 (ATF5), and CHOP is referred to as the integrated stress response (ISR) [31]. As PERK activity is highest in the pancreas, the PERK knockout mice experience rapid and progressive decline in endocrine and exocrine function, develop diabetes mellitus, show phenotypic abnormalities, and die shortly after birth [32,33]. Unlike the PERK knockout mice, the ATF4 knockout mice have no defects in glycemic control. However, the ATF4 knockout mice experience severe micro-ophthalmia with no other abnormalities [34]. An in vivo role of the PERK-ATF4 pathway that is causatively linked to DN has not been reported.

## 4. ATF6 Pathway

In addition to the IRE1 and the PERK pathways, UPR signaling is mediated by ATF6. ATF6α is a type II transmembrane protein that contains a bZIP transcription factor within its cytosolic domain. Upon activation, ATF6α (full length 90-kDa protein) is translocated to the Golgi apparatus, where regulated intramembrane proteolysis by the Site-1 and the Site-2 proteases liberates the N-terminal cytosolic portion, ATF6α-p50 [35,36,37,38]. The latter localizes to the nucleus, where it acts as a transcription factor, promoting the expression of gene encoding proteins that function to increase ER capacity and folding—including BIP, glucose regulated protein (GRP94 or Endoplasmin), p58IPK (also known as DNAJC3 and XBP1), as well as the ER-associated degradation (ERAD) components (Figure 1a) [39,40,41]. Both ATF6α-p50 and sXBP1 act alone or in a collaborative fashion to activate genes that are involved in mitigation of ER stress. Both ATF6α and ATF6β show a degree of functional redundancy and mice with a knockout of either ATF6α or ATFβ are viable. However, the combined deletion of ATF6α and ATFβ results in embryonic lethality [40]. Recent studies have demonstrated that the persistent activation of ATF6α drives pathogenic UPR in DN. Mice with the podocyte-specific inducible expression of ATF6α-p50 experience exacerbated features of streptozotocin induced DN [27].

Besides mediating UPR, stress sensors also function as scaffolding proteins, allowing physical interaction with various proteins, which modulate multiple cellular processes, including cytoskeleton dynamics, bioenergetics, signaling crosstalk with other stress pathways, inflammation, and cell differentiation [3,42,43]. Recent studies to this end have demonstrated that the components of insulin signaling, the regulatory subunits of PI3Kinase P85α and P85β, which lies downstream of insulin receptor (INSR) specifically drive sXBP1-dependent UPR to maintain cellular homeostasis (Figure 1a) [27,44]. Intracellular signaling intermediates mitogen activated protein kinase (p38 MAPK), forkhead box protein O1 (FOXO1) and IĸB kinase β (IKKβ), modulate XBP1 pathway in order to maintain glucose homeostasis [45,46,47]. Additionally, vascular endothelial growth factor (VEGF) signals via ATF6 and PERK to promote endothelial cell survival and angiogenesis [48]. Further characterization of the mechanisms underlying the homeostatic regulation of UPR might offer new therapeutic avenues for the treatment of DN.

## 5. Activation of UPR in Human DN

**Figure 2:**
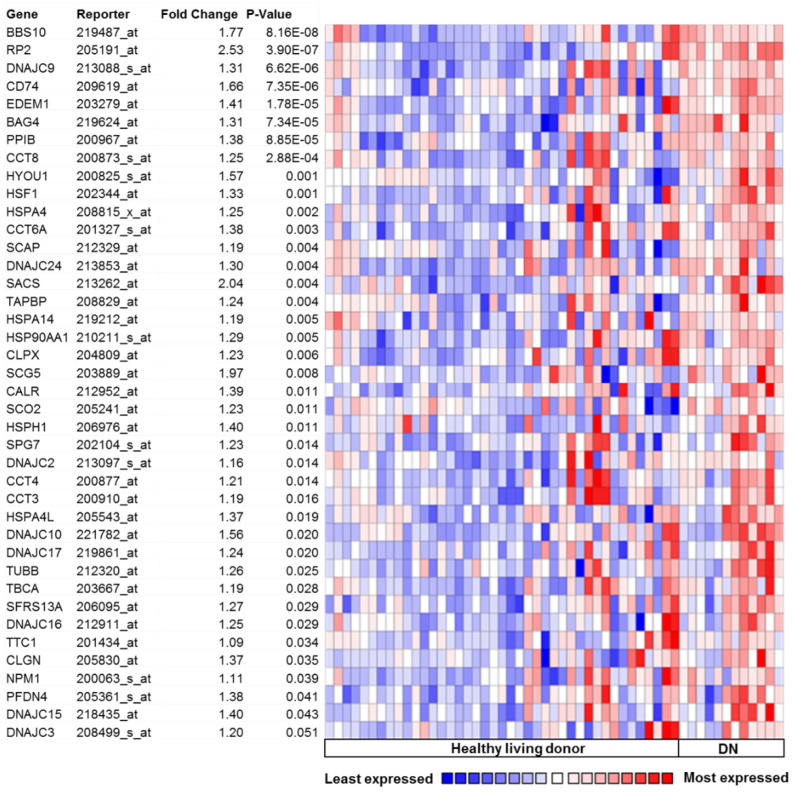
UPR regulating genes: Glomerular-specific gene expression in the Nephromine database (within “Ju podocyte” dataset). A large number of unfolded protein response genes are induced in human patients with well-established DN when compared to healthy controls (1: Healthy living donors (*n* = 41); 2: DN: diabetic nephropathy (*n* = 12); data queried for overexpression).

Chronic hyperglycemia and proteinuria in patients suffering from DN poses a greater challenge for the maintenance of ER-proteostasis. Interrogation of the microarray data from human renal biopsies obtained from the patients with established DN revealed significant differences in the genes regulating UPR, when compared to healthy human biopsies [49]. Even mild DN is associated with significant change in the expression of highly conserved ER-transcription factor XBP1 and the ER-chaperones, heat shock protein family A (HSPA5 or GRPR78/BIP), and hypoxia up-regulated 1 (HYOU1), indicating an association between the degree of UPR and DN. Morphological analysis in independent cohorts of patients revealed a higher expression of HSPA5 and HYOU1 in the tubular epithelia [27,49]. Together with the fenestrated glomerular endothelial cells (GECs) and glomerular basement membrane (GBM), podocytes form the glomerular filtration barrier. Disruption of the glomerular filtration barrier, associated with podocyte foot process effacement and loss of podocytes is a characteristic feature of DN [50,51,52]. In order to determine the pathophysiological relevance of UPR in DN and to identify the candidate pathways, we queried the public database Nephroseq (www.nephroseq.org), accessed date 10 March 2015, formerly Nephromine, a large collection of gene expression profiles from murine models and human patients with and without DN [53]. Gene expression analyses revealed a large number of key UPR regulating genes significantly upregulated in patients with DN indicative of ER stress (Figure 2). Specifically, genes involved in protein binding, protein folding (molecular chaperones), heat shock response, secretion, and intracellular trafficking (*BBS10, DNAJC9, DC74, BAG4, PPIB, CCT8, HYOU1, HSPA4, CCT6A, SCAP, SACS, TAPBP, HSPA14, HSP90AA1, SCG5, SCO2, HSPH1, DNAJC2, CCT4, CCT3, HSPA4L, DNAJC17, TBCA, DNAJC16, TTC1, CLGN, PFDN4, DNAJC*) were significantly upregulated in DN samples when compared to healthy living donors. Intriguingly, expression of the transcription factor, heat shock transcription factor 1 (*HSF1*), which predominantly controls the heat shock response and the cytosolic UPR was significantly upregulated in patients with DN. Additional genes upregulated in DN include *CALR* (calcium binding protein), *HSPH1* (prevents denaturation of aggregated proteins), *DNAJC24* (stimulates the ATPase activity of several Hsp70-type chaperones), *EDEM1, CLPX, DNAJC10* (involved in protein degradation), *RP2, SPG7, TUBB, NPM1* (organelle biogenesis and maintenance), *SFRS13A* (constitutive and regulated mRNA splicing), and *DNAJC15* (prevents mitochondrial hyperpolarization). The transcription factor ATF6α-p50 alone can bind to ER stress response element-II (ERSE-II), whereas ATF6α heterodimerizes with sXBP1 to induce a distinct subset of unfolded protein response elements (UPRE). While ATF6α is solely responsible for the transcriptional induction of major ER-chaperons, ATFα-sXBP1 heterodimer induces ERAD components. The gene expression pattern in DN predominantly indicate upregulation of genes involved in protein binding, protein folding (chaperones), and heat shock response (DNAJC family and HSPA family, CCT family), whereas the induction of the components of ERAD is minimal (Figure 2). The components of ERAD are highly expressed in podocytes of both mouse and human kidneys, and they play an essential role in nephrin maturation and kidney glomerular filtration [54]. Suppressed ERAD by intraglomerular crosstalk between mesangial cells and podocytes causes podocyte injury in diabetic mice [55]. While these data indicate that DN is associated with chronic ER stress, additional investigations are required to specifically define the role of ATF6α and ATF6α-sXBP1 heterodimer-specific subsets of genes that are involved in homeostatic and pathogenic UPR regulation.

## 6. UPR in Experimental Models of DN

Interrogation of the human renal biopsies suggested chronic activation of UPR in DN. Accordingly, exposure of renal tubular epithelial cells to albumin and high glucose in vitro enhanced expression of genes involved in ER stress [49]. Free fatty acids and reactive oxygen species (ROS) are critically involved in the pathogenesis of type 2 diabetes, in particular the regulation of pancreatic cell survival. Treatment of podocytes with the palmitic acid induced ER stress, exemplified by the upregulation of ER-chaperone BIP and pro-apoptotic transcription factor CHOP. Gene silencing of the CHOP protects against palmitic acid induced apoptosis [56]. Downstream of CHOP, TRB3 is a kinase-like molecule that modifies cell survival and metabolism was found to be upregulated in the kidneys of type-1 and type-2 diabetic mice. Elevated ROS and palmitic acid associated with diabetic milieu promote the expression of TRB3 in a CHOP-dependent manner. The ROS augments the recruitment of CHOP to the proximal TRB3 promoter. The CHOP-TRB3 axis promotes podocyte production of MCP-1/CCL2, a chemokine that contributes to the inflammatory injury associated with DN [57]. Additional in vitro studies showed that the cannabinoid receptor 1 mediates palmitic acid induced ER stress and apoptosis in renal tubular epithelial cells [58]. In addition to tubular epithelial and podocytes, hyperglycemia, free fatty acids, and advanced glycation end products (AGEs) promote ER stress associated cell death in mesangial cells [56,59,60,61]. High glucose treatment induces cell surface expression of GRP78 (BIP) in mesangial cells. The membrane localized GRP78 interaction with the integrin-β1 activates the focal adhesion kinase (FAK) and the downstream PI3K/AKT signaling, which is required for high glucose induced extracellular matrix synthesis (ECM). The XBP1 can activate the phosphatase and tensin homolog (PTEN)/AKT signaling and thereby alleviate high glucose induced oxidative stress, ECM synthesis, and mesangial cell apoptosis [62,63]. In vivo in mouse models of streptozotocin-induced DN, enhanced renal cell apoptosis is associated with an increased expression of the pro-apoptotic ER stress markers CHOP, C-Jun NH2-terminal kinase (JNK), and caspase-12 [64,65]. The CHOP-deficient mice were protected from DN and age-associated albuminuria [66]. DN is associated with the abnormal activation of the mTOR complex (mTORC1), a kinase that senses nutrient availability in podocytes [67,68]. Podocyte-specific activation of the mTORC1 due to genetic deletion of the upstream negative regulator (PcKOTsc1) recapitulated several features of the DN associated with ER stress. Inhibition of ER stress protected against the podocyte phenotypic switch and podocyte loss in PcKOTsc1 knock out mice with the abnormal activation of mTORC1 [67]. The expression of a specific isoform of ER-localized protein, reticulon 1A (RTN1A), has been shown to be upregulated in multiple experimental models of chronic kidney disease (CKD), including in Tg26, a chronic kidney disease (CKD) model of human immunodeficient virus associated nephropathy (HIVIAN), and in db/db mice with endothelial nitric oxide synthase (eNOS) deficiency (db/db-eNOS^-/-^ mice). Analyses of the human CKD samples (Nephromine database) likewise showed a higher expression of the RTN1A in patients with DN, when compared to the healthy controls. Knockdown of RTN1A expression in vivo attenuated ER stress, proteinuria, and renal fibrosis in mice with unilateral ureteral obstruction and also in diabetic mice [69]. Recent studies demonstrated the essential role of the IRE1 branch of UPR in regulation of ER-proteostasis by regulating autophagy [25]. These studies provide substantial evidence that the ER stress induced cell death plays a critical role in the regulation of DN. However, until recently, the mechanism through which diabetic milieu promotes maladaptive UPR, which provokes ER stress mediated cell death in glomerular cells remained unclear. Using a combination of in vitro and in vivo studies in podocyte-specific deletion mutants, analysis of human renal biopsies and gene expression database of patients with established DN, we have demonstrated that the deranged ER-proteostasis network is at the core of DN in both humans and mice [27]. In mouse models of insulinopenic (streptozotocin-induced) diabetes or in insulin-resistant db/db mice, DN is associated with the selective reduction in nuclear translocation of ER-transcription factor sXBP1. The impaired nuclear translocation of sXBP1 is associated with elevated nuclear levels of the transcription factor ATF6α and the pro-apoptotic regulator CHOP indicating a disparate regulation of these two branches of UPR in DN (Figure 1b). Congruently, in glucose stressed cultured podocytes as well as in GECs, the nuclear levels of sXBP1 decline with a concomitant increase in the nuclear levels of ATF6α and CHOP. Within the glomerular cell types, podocytes express insulin receptor (INSR). Insulin signaling via INSR in podocytes selectively regulates the nuclear translocation of ER-transcription factor sXBP1. Insulin-dependent nuclear translocation of sXBP1 is dependent on the regulatory subunits of PI3Kinase, p85α, and p85β [27]. Mechanistically, insulin signaling mediated by INSR induces binding of the regulatory subunits of PI3Kinase, p85α, and p85β with the sXBP1. Insulin induced binding of the p85α and p85β with sXBP1 promotes the nuclear translocation of sXBP1 and subsequently sXBP1 dependent gene expression (Figure 1a). Podocytes are highly insulin sensitive and insulin signaling in podocytes constitutively maintains sXBP1-dependent homeostatic UPR [27,70]. Accordingly, attenuating insulin signaling by podocyte-specific genetic ablation of the INSR and the downstream signaling intermediates, p85α or p85β and XBP1 abrogates insulin dependent adaptive responses, which propagates ATF6- and CHOP-dependent maladaptive responses in mouse models of DN [27]. The augmented ER-proteotoxicity in mouse models of defective insulin signaling in podocytes was ameliorated when mice were treated with the chemical chaperone TUDCA [27]. Importantly, histological analyses of the human renal biopsy samples and gene expression analyses in patients with DN (nephromine database) revealed an impaired p85-sXBP1 pathway and augmented pathogenic UPR signaling, corroborating our findings in animal models and in in vitro studies [27]. These studies for the first time, not only dissected the regulation of pathogenic UPR in DN but also demonstrated the specific UPR pathways that are causatively linked to DN, providing a new understanding of UPR-ER signaling in DN.

## 7. ATF6 Activation Is Necessary and Sufficient to Promote DN

Intriguingly, the ER stress in DN does not involve the PERK pathway. Insulin resistance (IR) or hyperglycemia does not induce the phosphorylation of eIF2α or the activation of ATF4 in chronic DN samples. Induction of ER stress in cultured glomerular podocytes or in endothelial cells with higher concentrations of glucose impaired nuclear translocation of sXBP1 with a concomitant increase in the nuclear levels of ATF6, but not ATF4. The ATF6-dependent activation of the CHOP mediates hyperglycemia induced cell death in podocytes [27]. Additionally, XBP1 mRNA is induced by the ATF6 and the collaborative signaling of ATF6 and sXBP1 mediates the activation of gene expression that restores the adaptive UPR and inhibits ER stress [39]. However, the lower insulin levels or IR in DN diminishes the nuclear translocation of sXBP1, which may provoke sole ATF6-dependent signaling (Figure 1b). Podocyte-specific deletion of XBP1 or inducible expression of ATF6α-p50 in podocytes is not sufficient to cause proteinuria in a normal healthy mouse [27]. Nevertheless, in experimental models of chronic streptozotocin-induced diabetes, podocyte-specific deletion of XBP1 or inducible activation of ATF6 aggravated features of DN. These data indicate that both impaired function of the sXBP1 followed by the sustained activation of the ATF6 is required for the overt manifestation of DN [27]. While the lack of ATF4 activation indicates cell and tissue-specific regulation of UPR in diabetes. Alternatively, it is reasonable to hypothesize that the activation of ER stress in these experimental models of DN falls below the threshold that activates the PERK-eIF2α-ATF4 pathway. Additionally, in DN, the impaired activation of sXBP1 is not due to dysfunctional IRE1 mediated sXBP1 processing, but rather defective insulin-dependent (*via* INSR) activation of the p85α and p85β that mediates IRE1 independent, nuclear translocation of the sXBP1 (Figure 1a). While ATF6 activation triggers ER stress dependent cell-death in DN, the underlying mechanism by which the loss of sXBP1 function triggers ATF6 activation remains to be investigated. Activation of the ATF6 is dependent on its glycosylation status and a reduction of the cysteines within the luminal region [71]. In unstressed ER, ATF6 exists in monomer, dimer, and oligomer forms. This is due to the presence of intra- and inter-molecular disulfide bridges formed between the two conserved cysteine residues within the luminal domain of ATF6. Inhibition of glycosylation by tunicamycin promotes translocation of ATF6 monomer to the Golgi complex for further limited proteolysis by site-1 and site-2 proteases (S1P and S2P), resulting in the generation of a highly active ATF6-p50 transcription factor. Additionally, the stability of ATF6 is regulated by sXBP1 target gene wolfram syndrome 1 (WFS1) through the proteasome-mediated degradation [72]. These studies demonstrate the critical role of ATF6 in progression of DN. However, the mechanism that controls the ATF6 activation in chronic DN requires further investigation.

## 8. XBP1 Integrates Insulin and Coagulation Protease Signaling

**Figure 3:**
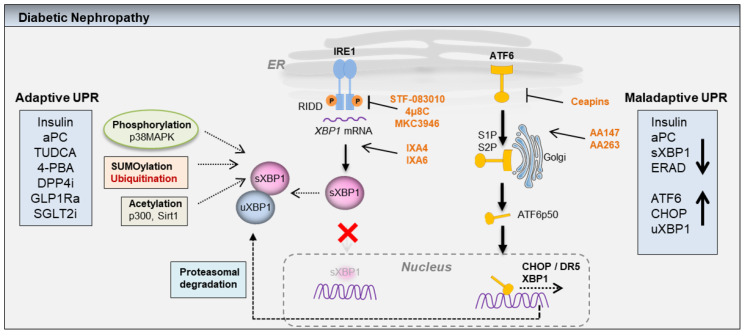
Potential mechanisms that modulate UPR in diabetic nephropathy (DN): post-translational modifications such as phosphorylation (p38MAPK), SUMOylation, ubiquitination, and acetylation regulate stability, nuclear translocation, and transcriptional activation of sXBP1. In DN, loss of insulin or aPC dependent adaptive UPR mediated by sXBP1 or ER-associated degradation (ERAD) deficiency leads to sustained activation of maladaptive ATF6/CHOP signaling. In addition to the activation of proapoptotic CHOP and DR5, ATF6 can potentially induce XBP1 expression, and the binding of unspliced XBP1 (uXBP1) to spliced XBP1 (sXBP1) mediates proteasomal degradation of the transcriptionally active sXBP1. However, a role for the ATF6-uXBP1 pathway in regulation of proteasomal degradation of sXBP1 in DN remains to be investigated. In addition to hemostatic UPR regulators insulin and aPC, the nephroprotective pharmacological compounds including TUDCA, 4-PBA, DPP4 inhibitors (DPP4i), GLP1R agonists (GLP1Ra), and SGLT2 inhibitors (SGLT2i) may modulate adaptive UPR. Small molecule compounds STF-083010, 4µ8C, MKC3946 are IRE1α-RNase inhibitors, whereas IXA4 and IXA6 selectively activate IRE1α-dependent sXBP1 activation. Ceapins selectively inhibit ATF6α activation, whereas compounds AA147 and AA263 target protein disulphide isomerases (PDIs) to selectively induce ATF6α activation.

DN is a microvascular disease associated with increased coagulation activation as a result of impaired endothelial thrombomodulin (Thbd)-dependent protein C (PC) activation in both human subjects and mice [73]. The Thbd is an endothelial glycoprotein predominantly expressed on the vascular endothelium. Coagulation activation and the increased generation of thrombin promotes formation of thrombin-Thbd complex on the endothelial cell surface. The Thrombin-Thbd complex obtains substrate specificity for the zymogen PC, and it generates activated protein C (aPC). The aPC generated through this process not only mediates anticoagulant function, but also exerts anti-inflammatory and cytoprotective effects on endothelial cells and podocytes [74]. Thus, aPC inhibits hyperglycemia induced mitochondrial dysfunction in microvascular endothelial cells, and it protects against both endothelial and podocyte apoptosis in animal models of DN [73]. Multiple cell-specific receptor complexes mediate the protective functions of aPC [75]. On endothelial cells, the cytoprotective aPC signaling requires endothelial protein C receptor (EPCR) and protease activated receptor-1 (PAR1) [74,76]. However, in podocytes, the cytoprotective effects of aPC are mediated by a distinct mechanism that involves integrin-α_v_β_3_ and protease activated receptor-3 (PAR3) [77]. Akin to insulin, aPC regulates the nuclear translocation of transcription factor sXBP1 to maintain UPR. Disruption of aPC or insulin signaling both impairs the function of podocytes and ultimately causes dysfunction of the glomerular filtration barrier, promoting DN [27,78]. Both, insulin and aPC converge on a common sXBP1 signaling pathway to maintain ER homeostasis. Importantly, in mice with podocyte-specific genetic deficiency of the INSR, aPC selectively restores the activity of the cytoprotective ER-transcription factor sXBP1 by temporally targeting INSR downstream signaling intermediates, the regulatory subunits of PI3Kinase, p85α, and p85β [78]. Both insulin and aPC independently modulate UPR by selectively activating sXBP1, thereby inhibiting ER stress associated cell death mediated by the ATF6-CHOP axis. While the podocyte-specific activation of ATF6α is sufficient to promote DN, the loss of the sXBP1 function is an upstream event that is critical for the maintenance of homeostatic-UPR in podocytes [27]. The function of sXBP1 can be regulated by various post translational modifications (PTMs), which includes phosphorylation, SUMOylation, ubiquitylation, and acetylation (Figure 3). The SUMOylation of XBP1 reduces its transcriptional activity but not its nuclear translocation [79], whereas p38MAPK regulates the phosphorylation of XBP1 at the amino acid residues, threonine48 (Thr48) and serine68 (Ser68), which enhances its nuclear translocation [45]. The ubiquitination of sXBP1 at lysine residues (K60 and K77) mediates its proteasomal degradation. The double mutants (K60/77R) with higher resistance to ubiquitination showed increased nuclear translocation and enhanced transcriptional activity [80]. Additionally, histone acetyl transferase p300 (p300 HAT) and sirtuin1 (SIRT1) modulate the acetylation of sXBP1. While p300 regulates the acetylation, stability, and transcriptional activity of sXBP1, SIRT1 deacetylates sXBP1, inhibiting its transcriptional activity [81]. The unspliced form of XBP1 (uXBP1) is unstable and rapidly degraded following ubiquitination by the proteasome because of the degradation motif contained within the uXBP1. Additionally, uXBP1 when bound to sXBP1 targets sXBP1 for proteasomal degradation, thus acting as a negative regulator of sXBP1(Figure 3) [82]. This mechanism may be beneficial to shut off sXBP1-dependent acute responses. However, how the uXBP1 mediated proteasomal degradation or other PTMs such as SUMOylation, phosphorylation, ubiquitination, and acetylation modulate the function of sXBP1 in a chronic disease like diabetes warrants additional investigation. While the PTMs of XBP1 modulates its function, sXBP1 triggers the activation of the hexosamine biosynthetic pathway (HBP). In experimental models of ischemia/reperfusion injury of hearts, sXBP1 induces transcriptional activation of multiple enzymes of HBP. The rate limiting enzyme of HBP, glutamine fructose-6-phosphate aminotransferase 1 (GFAT1), is a direct target of the sXBP1. Accordingly, sXBP1 overexpression in vivo significantly enhances HBP flux and O-linked coupling of N-acetylglucosamine (O-GlcNAc) [83]. Post-translational modification of proteins by the O-GlcNAc is a dynamic process that governs the function of numerous cytosolic and nuclear proteins [84]. Increased levels of O-GlcNAc protein modifications contribute to the pathogenesis of diabetes and other diseases such as cancer and neurodegenerative diseases [85]. Therefore, studies focusing on the mechanisms that preserve or enhance the sXBP1 function or on altered pathways downstream of sXBP1 signaling that may propagate DN, are needed.

## 9. Pharmacological Targeting of UPR in DN

Nephropathy develops in approximately 35% of patients with type 2 diabetes, and it is associated with increased mortality. Intensive glucose-lowering strategies and the use of single-agent blockade of the renin-angiotensin-aldosterone system (RAAS) have been shown to reduce surrogate markers of renal complications in patients with type 2 diabetes. However, the patients with type 2 diabetes remain at increased risk for death due to cardiorenal complications [86]. The correlative analysis of ER stress markers in renal tissues from human patients together with studies using genetic manipulation of the UPR in experimental models of DN suggest that maladaptive UPR signaling makes a direct contribution for the pathogenesis of DN. The critical role of UPR signaling in multiple metabolic and non-metabolic diseases including diabetes, obesity, cancer, neurodegeneration, and immune-related conditions fostered development of several small molecule modulators of UPR [87]. Given the disparate roles of the XBP1 and the ATF6 branches of UPR in DN, within this section we will discuss the relevant therapeutic approaches that modulate these two branches of UPR.

## 10. ATF6 Modulating Compounds

Although the two closely related homologs ATF6α and ATF6β act redundantly during development, they do not appear to act redundantly during ER stress as ATF6α knockout cells or animals die when challenged with ER stressors [40]. Gene inactivation of ATF6α in mouse, fish, and cultured human cells have revealed the dominant role of ATF6α in regulating the expression of core UPR targets, such as major classes of ER chaperones: BIP, its cofactors, GRP94, and protein disulphide isomerases (PDIs) [88]. Whereas the role and the transcriptional targets of ATF6β during ER stress remain poorly understood. Chemical inhibition of S1P interferes with ATF6 activation [89]. However, inhibition of S1P also affects the proteolytic processing of SREBP (a cholesterol-regulated transcription factor) and other housekeeping proteins causing pleotropic effects. Using cell-based screens with ER stress-responsive luciferase reporters, both small molecule agonists and antagonists have been discovered for ATF6α. Screening more than 100,000 compounds for their ability to reduce ATF6α regulated genes yielded specific inhibitors for ATF6α named Ceapins [90]. Ceapins are a class of isoxazole ring-containing pyrazole amides that do not affect signaling through the other branches of the UPR, or proteolytic processing of its close homolog ATF6β or SREBP, both activated by the same proteases. Mechanistically, Ceapins are thought to block transport of ATF6α from the ER to the Golgi apparatus during ER stress [91]. In particular Ceapin-A7 has been shown to block induction of ATF6α transcriptional targets at submicromolar concentrations [90]. The utility of Ceapins as tool compounds has so far been proven in cultured mammalian cells. Additional studies in specific renal cell types and in experimental models of DN are required to evaluate the efficacy of these compounds in vivo. Selective ATF6α inducers (compounds 147 and 263) were discovered using a similar system. The compound 147 (AA147) is metabolically activated and covalently modifies ER resident PDIs to specifically induce ATF6α, whereas the mechanism of action of the compound 263 (AA263) remains to be established [92]. In striking contrast to the role of ATF6α in chronic DN, pharmacological activation of ATF6α using compound 147 in acute ischemia/reperfusion injury of heart transcriptionally reprograms proteostasis, ameliorates damage and preserves heart function [93]. Genetic inactivation of ATF6α specifically in cardiomyocytes abolishes the cardioprotective effect mediated by compound 147 indicating the critical role of the ATF6α-dependent proteostasis pathway. Likewise, pharmacological activation of the ATF6α pathway also protected against renal and cerebral ischemia/reperfusion injury in mice [93]. The opposing effects of ATF6α activation in chronic models of DN and in acute renal injury suggests that the initial activation of the ATF6α pathway aims to restore proteostasis, however, unresolved ER stress and persistent activation of ATF6α may propagate CHOP-dependent maladaptive responses during progression of DN.

## 11. IRE1/XBP1 Modulating Compounds

In addition to the processing of XBP1 splicing, IRE1α mediates additional functions through RIDD and through activation of TRAF2/JNK signaling (Figure 1b). Thus, IRE1α activation exerts both protective and harmful effects in homeostatic and disease-specific conditions. In mouse models of atherosclerosis, small molecule compounds that inhibit IRE1α ameliorate metabolic inflammation and counteract the progression of the disease [94]. Likewise, IRE1α endoribonuclease domain inhibitor MKC-3946 alleviates aortic dissection by decreasing vascular smooth muscle cell apoptosis [95] (Figure 3). The loss of the XBP1 function in diabetes may enhance IRE1α activity. However, the specific contributions of IRE1α-RIDD axis and IRE1α/sXBP1 axis in DN remains to be elucidated. Small molecule compounds IXA4 and IXA6 selectively activate the IRE1α-sXBP1 dependent proteostasis pathway without globally activating UPR and other stress-responsive signaling pathways (for example, heat shock response or oxidative stress response) [96]. Pharmacological administration of IXA4 transiently activates IRE1α/sXBP1 signaling in the liver without inducing RIDD or TRAF2/JNK signaling. In a mouse model of diet-induced obesity, IXA4 administration improves systemic glucose metabolism and liver insulin action, and it enhances the pancreatic function [97]. Given the crucial role of sXBP1 in the regulation of DN, it would be a promising approach to test such compounds in preclinical models of DN.

## 12. Glucose Lowering Compounds

Several compounds that are currently in use or that are being developed for the treatment of diabetes include glucose lowering agents and compounds that combat IR. These include sodium-glucose-co-transporter inhibitors (SGLT) and dipeptidyl-peptidase 4 (DPP4) inhibitors. The SGLT inhibitors reduce hyperglycemia by reducing renal absorption of glucose, thereby increasing urinary glucose excretion. Lowering glucose levels with the SGLT2 inhibitor (dapagliflozin) attenuated ER stress and significantly reduced albuminuria in mice [27]. In patients with type 2 diabetes, who were at higher cardiovascular risk, treatment with specific SGLT2 inhibitor empagliflozin delayed progression of DN associated with lower rates of clinically relevant renal events [98]. Further analysis from the EMPA-REG OUTCOME trial indicates empagliflozin may assist to prevent DN progression in patients with type 2 diabetes, irrespective of the common background medications that alter internal hemodynamics [99]. In addition to the consequences of profuse glycosuria and natriuresis, SGLT2 inhibition influences the central metabolic pathways of lipid oxidation and ketogenesis at the expense of carbohydrate utilization. Abnormal glucose utilization in diabetes may promote mitochondrial dysfunction and renal fibrosis. The key glycolytic enzyme pyruvate kinase M2 (PKM2) can be aggregated into tetrameric and dimeric forms. Hyperglycemia and diabetes decrease PKM2 tetramer formation and activity by sulfenylation in moue glomeruli and cultured podocytes. Genetic inactivation of PKM2 in podocytes exacerbated DN. Conversely, pharmacological intervention studies in DBA2/J and Nos3 (eNOS) deficient mice with a small molecule compound (TEPP-46), which specifically activates PKM2 reversed metabolic abnormalities, mitochondrial dysfunction, and protected against DN [100,101]. Mechanistically, loss of sirtuin 3 (SIRT3) in diabetic mice is associated with the induction of transforming growth factor-β (TGF-β)/smad signaling, higher levels of hypoxia-inducible factor1α (HIF1α) accumulation, and PKM2 dimer formation. The PKM2 dimer can enter into the nucleus to regulate gene expression which contributes to epithelial-mesenchymal transition and renal fibrosis [102]. Reducing blood glucose levels with SGLT2 inhibitors may influence systemic levels of hormones insulin, glucagon, and gastrointestinal peptides such as glucagon-like peptide1 (GLP1). Importantly, insulinotropic gastrointestinal hormone GLP1 plays an essential role in the regulation of insulin production and in insulin sensitivity in various metabolic organs including the kidney. Recently several DPP4 inhibitors that inhibit or delay the degradation of GLP1 have been considered as additional therapy for patients with DN [103]. Both GLP1R agonists and DPP4 inhibitors reduce the onset and the progression of albuminuria in patients with type 2 diabetes [104]. Of note, specific angiotensin converting enzyme inhibitor (ACEi), but not angiotensin II receptor blocker (ARB), ameliorated renal fibrosis by mitigating DPP4 and TGF-β signaling [105]. These studies indicate that the therapies that mitigate IR or restore optimal glucose metabolism in glomerular cells may have beneficial outcomes on top of traditional ACE inhibitors which are first-line medications for the treatment of DN [106]. In addition to the regulation of insulin sensitivity and presumably insulin-dependent homeostatic UPR, several compounds, for examples GLP1R agonists can exert pleotropic effects as GLP1R is widely expressed in various cell types including renal and immune cell types. Experimental studies indicate that modulation of systemic and local inflammation by GLP1R agonists contribute to the beneficial outcome in DN [104]. Therefore, future studies investigating the impact of these nephroprotective compounds on regulation of UPR and ER stress will provide additional insight into the role of renal cell proteostasis in DN.

## 13. Future Perspectives

Accumulating evidence in DN and in other chronic disease models indicate that the regulation of UPR in a disease state does not involve activation of all the three major pathways. For example, our studies in experimental models of diabetes showed that the PERK/ATF4 pathway is not activated during the progression of DN. Contrarily, ATF6 activation appears to play a critical role in the progression of DN [27,78]. Congruent with these observations, lower concentrations of ER stress inducing agents failed to elicit a PERK-dependent cell death program [107]. Additionally, critical components of the UPR, IRE1, and PERK are low abundant proteins and their abundance and intracellular localization within the specific ER subdomains may vary depending on the cell type. Therefore, it is possible that the disease-specific stimuli and the altered microenvironment within the renal tissue may result in distinct sensing and activation of the UPR. Recent studies indicate that the intraglomerular crosstalk between glomerular mesangial cells and podocytes suppresses ER-associated degradation process and causes podocyte injury in DN [55]. Podocyte–endothelial crosstalk mediated through glucocorticoid receptors is important for glomerular homeostasis [108,109]. Therapeutic interventions in animal models of DN using chemical chaperones TUDCA and 4-PBA, normalizing hyperglycemia using SGLT2 inhibitors, or recent studies using anti-sense oligonucleotides for CHOP show that alleviating ER stress halts or partially reverses the progression of DN. Inhibition of ER stress in these pre-clinical models of diabetes demonstrated additional benefit in preserving the renal function on top of ACE inhibitors, which are currently in use for the treatment of patients suffering from DN [110,111,112]. Chemical chaperone TUDCA is a ligand for both the farsenoid X receptor (FXR) and the G-protein coupled bile acid receptor (GPBAR1 or TRG5), whereas 4-PBA inhibits histone deacetylases (HDACs). Both TUDCA and 4-PBA are currently in clinical trials for amylotropic lateral sclerosis and cystic fibrosis, respectively [113]. Additionally, strategies to improve the activity of sXBP1 have shown beneficial effects in experimental models of obesity [97,114]. We opine that the field is only emerging and future studies in disease models that closely resemble human DN (for example in eNOS^-/-^ deficient mice) may reveal additional mechanisms that will enable researchers to develop therapies targeting maladaptive ER stress responses in DN.

## Data Availability

Not applicable.

## Abbreviations

ATF6	Activating transcription factor 6
ATF4	Activating transcription factor 4
CHOP	CEBP homologous protein
IRE1	Inositol requiring enzyme 1
PERK	PKR-like ER Kinase
DN	Diabetic Nephropathy
ER	Endoplasmic reticulum
ERAD	ER-associated degradation
RIDD	Regulated IRE1 dependent decay
UPR	Unfolded protein response
XBP1	X-box binding protein-1
